# Nutritional Deficiencies Following Bariatric Surgery: A Rapid Systematic Review of Case Reports of Vitamin and Micronutrient Deficiencies Presenting More Than Two Years Post‐Surgery

**DOI:** 10.1111/cob.70035

**Published:** 2025-07-28

**Authors:** Sophie Haughton, Sarah Gentry, Helen M. Parretti

**Affiliations:** ^1^ Sheffield Teaching Hospitals, Northern General Hospital Sheffield UK; ^2^ Norwich Medical School University of East Anglia Norfolk UK

**Keywords:** bariatric surgery, deficiency, mineral, nutrition, post‐surgery, vitamin

## Abstract

Bariatric surgery is the most clinically‐ and cost‐effective intervention for severe obesity. However, without adequate follow‐up, it can lead to nutritional deficiencies. Patients require life‐long nutritional supplements and follow‐up to prevent nutritional deficiencies from developing. This rapid systematic review is the first synthesis of case reports of patients with vitamin and micronutrient deficiencies at least 2 years following bariatric surgery, the point at which patients are typically discharged from specialist bariatric services. Eighty‐three cases (74 studies) met inclusion criteria. Female patients accounted for 84% of the reports. Roux‐en‐Y Gastric Bypass (RYGB) was the most common procedure to have been performed, followed by biliopancreatic diversion (BPD). The most frequently reported deficiencies were vitamin A (*n* = 15), copper (*n* = 14) and vitamin D (*n* = 23). In some cases, vitamin replacement led to symptom resolution, but some preceded permanent disability or death. Fifty‐one case reports detailed factors contributing to the development of the deficiency. These could be divided into patient factors or health care factors and provide areas to target interventions, including support to adhere to supplementation, appropriate follow‐up, and health professional awareness.

**Summary**

- What is already known about this subject?
  ○ Bariatric surgery is the most clinically‐ and cost‐ effective intervention for severe obesity.
  ○ Micronutrient deficiencies are some of the most common complications following bariatric surgery.
  ○ Patients require life‐long nutritional supplements and follow‐up to prevent nutritional deficiencies developing after bariatric surgery.
- What this study adds?
  ○ This rapid systematic review of case reports synthesises current evidence on micronutrient deficiencies presenting two or more years after bariatric surgery.
  ○ Included case reports highlight missed opportunities for earlier intervention, including lack of appropriate nutritional counselling, follow‐up and support to adhere to supplementation.
  ○ Health professionals must be aware of late micronutrient deficiencies in patients with a history of bariatric surgery.

## 1 Introduction

Obesity is a significant public health problem. In 2022, 1 in 8 people in the world were living with obesity [1]. In England, the prevalence of obesity among adults is 25.9% [2], and in the USA, the prevalence of obesity in people aged 20 or older is 41.9%. Obesity is associated with increased morbidity and mortality, greater use of healthcare resources, and adverse impacts on wider economic development [3, 4]. Bariatric surgery is the most clinically‐ and cost‐effective intervention for severe obesity [5, 6]. Its benefits extend beyond weight loss and include remission of type two diabetes, improvements in hypertension, and reductions in cardiovascular disease [5, 7]. However, it can be associated with complications, including nutritional deficiencies. Micronutrient deficiencies are some of the most common complications following bariatric surgery [8]. They can occur many years after surgery, posing a challenge to identification and appropriate management.

In England in 2021, 2022, 4035 people had primary bariatric surgery funded by the National Health Service [9]. The European Association for the Study of Obesity (EASO) guidance advises that post‐operatively, patients should be followed up lifelong [10]. They recommend patients should be routinely given micronutrient supplementation and indefinitely screened for nutritional deficiencies [8, 10]. The type of bariatric procedure undertaken determines which nutrients are recommended, which deficiencies should be screened for and how regularly this screening should take place. In England, the National Institute for Health and Care Excellence (NICE) guidance advises that patients stay under follow up from specialist bariatric services for a minimum of 2 years [6]. After this, patients should be discharged to primary care for annual monitoring of nutritional status with appropriate supplementation under a shared care model.

Patients may not be receiving adequate long‐term follow‐up after discharge from specialist bariatric services. A cohort study of post‐bariatric surgery nutritional follow‐up suggested that if specific blood tests were used as a proxy for annual bariatric surgery review, only around 5% of patients were receiving appropriate long‐term follow‐up in primary care [11]. Contributing factors include a lack of access to specialist services, a lack of education, training, and support for both patients and primary care staff, funding, and difficulties for patients complying with medications (including remembering to take them, side effects and cost) [5, 12, 13]. This leaves patients vulnerable to nutritional deficiencies, which may have a significant impact on a patient's health trajectory [14]. A systematic review and meta‐analysis (*n* = 54 articles) on nutritional deficiencies at long‐term follow‐up post‐bariatric surgery (5–17 years) found that the most common deficiencies were vitamin D (35.8%), vitamin E (16.5%), vitamin A (13.4%), vitamin K (9.6%) and vitamin B12 (8.5%) [15]. Prevalence of vitamin A and folate deficiencies increased with the follow‐up time. In a systematic review and meta‐analysis (*n* = 8 studies) of patients followed up long‐term (at least 5 years) after biliopancreatic diversion with duodenal switch, 25.4% of patients had a vitamin A deficiency, 57.3% had a vitamin D deficiency, 22.2% had a calcium deficiency, 69.7% had abnormal parathyroid hormone levels, and 29% had abnormal ferritin levels [16]. These demonstrate that multiple late nutritional deficiencies are common post‐bariatric surgery.

Synthesising case reports may provide details on the sequelae for real patients of nutritional deficiencies and highlight issues around follow‐up, factors contributing to deficiencies, correct recognition of deficiencies by clinicians, and variability in treatment. This rapid review aims to provide a greater understanding of the circumstances in which micronutrient deficiencies two or more years after bariatric surgery may occur, their consequences for individual patients, and factors contributing to these deficiencies to help direct future intervention efforts.

## 2 Materials and Methods

A rapid systematic review of case reports and case series of patients who developed micronutrient deficiencies 2 years or more post‐bariatric surgery was conducted in accordance with the Preferred Reporting Items for Systematic Reviews and Meta‐analyses (PRISMA) guidelines [17].

A rapid review approach was taken, as described by Khangura et al. [18]. This provides robust evidence summaries informing policy and practice, incorporating key aspects of systematic review methodology, but with results delivered in a timeframe appropriate for decision‐makers.

### 2.1 Inclusion Criteria

#### 2.1.1 Study Design

Case reports, or case series (more than 4 cases [19] and differentiated from cohort studies according to Mathes et al. [20]). These were chosen as we were seeking individual‐level data to understand trajectories and factors influencing late micronutrient deficiencies. Cases included in a wider paper for example, accompanying a literature review were included. If a paper included multiple cases, cases were individually assessed against our eligibility criteria and those meeting our criteria were included.

Although a systematic review of case reports cannot assess the incidence of micronutrient deficiencies after bariatric surgery or causality between risk factors and outcomes, it can collate individual‐level trajectories and generate hypotheses for subsequent studies.

### 2.2 Participants

Adults (≥ 18 years) who have undergone bariatric surgery and experienced post‐surgical deficiency of one or more of the micronutrients outlined in national guidance as requiring monitoring [21].

Bariatric surgery included gastric bypass, gastric band, BPD and duodenal switch, sleeve gastrectomy and any other recognised form of bariatric surgery. The bariatric surgery must have been performed ≥ 2 years ago, as this is when guidelines recommend that patient care should be transferred from specialist services to primary care [21]. Studies were included from any country.

#### 2.2.1 Exclusion Criteria

(1) Papers not available in the English language. (2) Papers that were conference abstracts, letters, opinion pieces, or editorials. (3) Reports including patients whose obesity was caused by genetic disorders and other syndromes. (4) Reports focusing on surgery for reasons other than weight loss. (5) Reports focusing on revisional or reversal of bariatric surgery rather than primary bariatric surgery. (6) Reports focusing on protein deficiencies as the main nutritional deficiency. (7) Reports not stating the type of bariatric surgery nor the time since the surgery. (8) Reports not including enough data to extract or to perform a quality assessment.

#### 2.2.2 Search Strategy

MEDLINE and EMBASE databases were searched via Ovid from January 2000 until January 2024. The search strategy was devised to find case reports focusing on any nutritional deficiencies experienced by patients at least 2 years after bariatric surgery. Search terms were tailored where necessary to the database search in order to maximise the capture of relevant papers. EndNote was used for reference management. An example of the search strategy used for the MEDLINE search can be found in Appendix 1.

#### 2.2.3 Study Screening, Data Extraction and Quality Appraisal

After de‐duplication, titles and abstracts were screened according to pre‐specified inclusion/exclusion criteria by 1 author (SJH/SVG) with 10% double screened by a second (SJH/SVG/HMP). Uncertainties were resolved by discussion. Potentially included full text articles were retrieved and reviewed, and 10% double screened.

Data were extracted using a predesigned proforma developed by the authors and quality assessed using The Joanna Briggs Institute Critical Appraisal tool for case reports and case series by a single reviewer [22] (SJH/SVG). We did not assess according to the criteria “Were adverse events (harms) or unanticipated events identified and described?” as our outcome of interest was an adverse event and so this criterion would not differentiate quality between our included studies. 10% of data extractions and critical appraisals were checked by a second reviewer (SJH/HMP). Double screening and data extraction of only a sample was necessary due to resource limitations, and has been done in similar reviews [23, 24, 25, 26].

A narrative synthesis was planned from the protocol stage, based on guidance by Popay et al. [27].

## 3 Results

**FIGURE 1:**
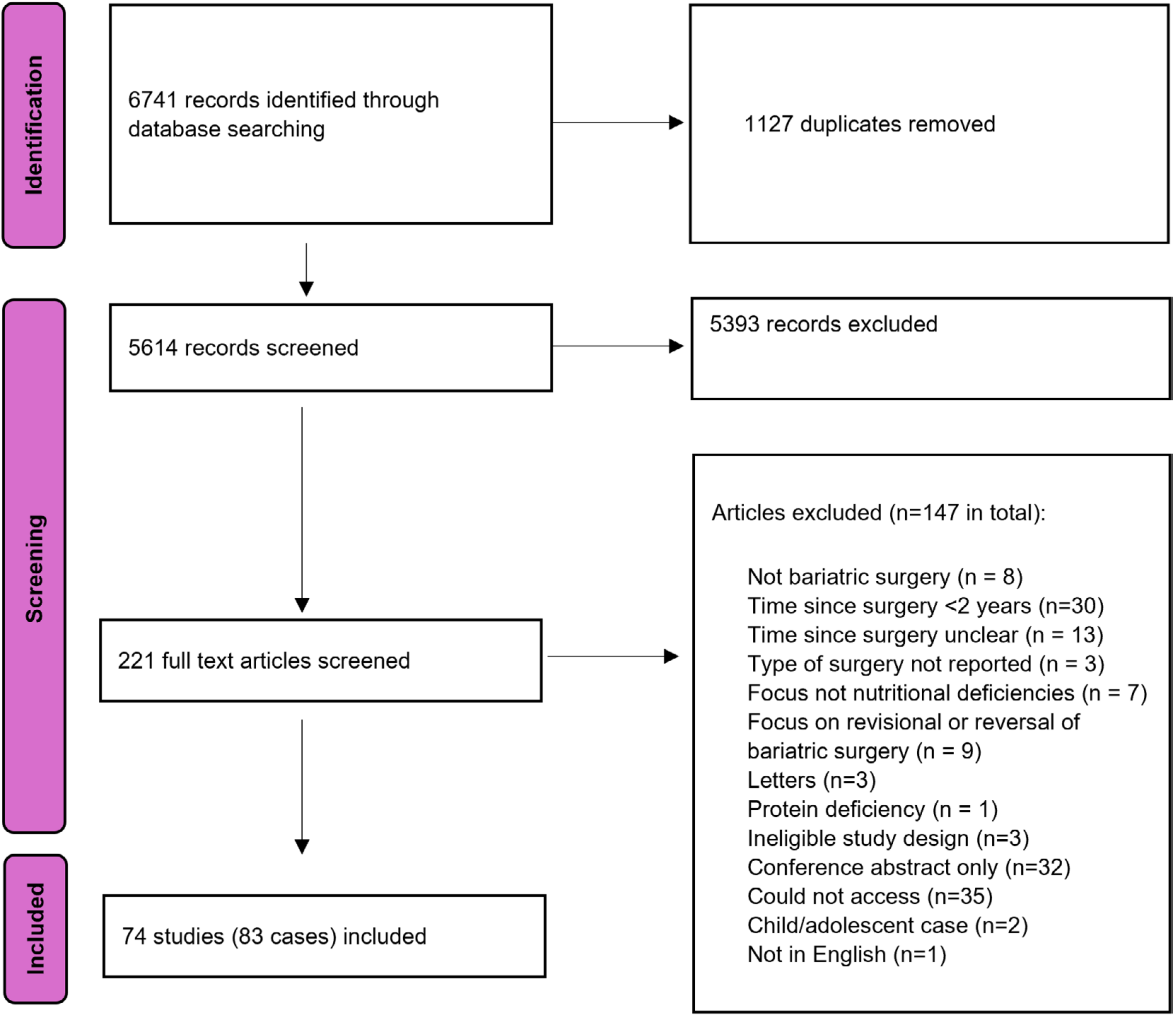
PRISMA flow diagram.

The PRISMA Flow Diagram [17] reports records identified, duplicates, records screened and included/excluded, full‐text articles assessed, and studies included in narrative synthesis (Figure 1).

### 3.1 Study Characteristics

Searches revealed 83 cases meeting the inclusion criteria, reported in 74 articles (Table 1).

**TABLE 1:** Characteristics of included studies.

Study ID	Case ID	1st author and year	Procedure	Years ago	Deficiency	Signs, symptoms and diagnosis	Management	Sequalae	Factors contributing
1	1	AlHassany 2014 [28]	SG; DS	4	Copper; Vitamin A	Night blindness and paraesthesia	Medication: copper, vitamin A, folic acid and iron	Remained night blind	Diarrhoea
2	2	Alkurdi 2021 [29]	Jejunoileal bypass	40	Vitamin A, Vitamin C, Vitamin D, Vitamin B6	Weight loss, diarrhoea, reduced general status	Medication: Enteral and parenteral nutrition with trace elements and vitamins; high dose vitamin E	Improvement	Only taking calcium and folic acid supplements prior to presentation
3	3	Al‐Shoha 2009 [30]	BPD	4	Vitamin D	Osteomalacia	Medication: ergocalciferol and calcium carbonate	Improvement	Misdiagnosis Inadequate doses
3	4	Al‐Shoha 2009 [30]	RYGB	5	Vitamin D	Osteomalacia	Medication: ergocalciferol and calcium carbonate	Improvement	Misdiagnosis Inadequate doses
3	5	Al‐Shoha 2009 [30]	RYGB	5	Vitamin D	Osteomalacia	Medication: ergocalciferol and calcium carbonate	Improvement	Misdiagnosis Inadequate doses
4	6	Amin 2022 [31]	Roux‐en‐Y gastric bypass	20	Copper	Sensory ataxia and neuropathy	Medication: copper	Improvement	Delayed diagnosis
5	7	Atreja 2003 [32]	Partial gastrectomy with BPD; DS	12	Vitamin D	Osteomalacia	Medication: calcium, calcitriol, vitamin B12; ergocalciferol	Improvement	Misdiagnosis
6	8	Bae‐Harboe 2012 [33]	Gastric bypass	3	Zinc	Skin pathology	Medication: zinc	Improvement	NR
7	9	Barro 2017 [34]	RYGB	18	Vitamin D	Osteomalacia	Surgery: for fractures Medication: calcium, vitamin D, vitamin B12, ferrous sulfate, multivitamins	Resolution	LTFU
8	10	Beh 2013 [35]	Gastric band	6	Thiamine	Wernicke's Encephalopathy	Medication: thiamine and folate	Resolution	Alcohol excess Vomiting
9	11	Benhalima 2009 [36]	BPD	2	Vitamin D	Brown tumour of bone	Surgery: tumour removal Medication 25‐OHD, calcium	Resolution	LTFU Nonadherent to supplementation
10	12	Bergmann 2020 [37]	Gastric bypass	6	Iron	Anaemia	Medication: iron	Resolution	NR
11	13	Bétry [38]	Gastric bypass	> 3	Vitamin B9, Vitamin A, Selenium	Vomiting, nutritional complications and an anastomotic ulcer	Surgery: conversion to Roux‐en‐Y for ulcer Medication: artificial nutrition	NR	NR
11	14	Bétry [38]	Gastric band	13	Vitamin B6, Vitamin A, Selenium, Zinc	Encephalopathy, vomiting, malnutrition	Medication: parenteral nutrition Surgery: removal of band	Sepsis from PN, PN stopped after band removal	NR
12	15	Bowley 2017 [39]	RYGB	4	Copper	Myelopathy	Medication: copper chloride, copper, pregabalin, gabapentin	Improvement	Micronutrient interaction (zinc)
13	16	Brancatella 2017 [40]	BPD	20	Vitamin D	Osteomalacia (incidental finding)	Medication: albumin, calcium gluconate, calcitriol, calcium carbonate, calcifediol Other: calorie intake increased	Improvement	LTFU Deficiencies previously found but not acted upon Poor oral nutrition
14	17	Breazzano 2023 [41]	BDP with duodenal switch	13	Vitamin A	Night blindness and blurred vision	Medication: vitamin A	Resolution	Nonadherent to supplementation
14	18	Breazzano 2023 [41]	BPD	15	Vitamin A	Poor night vision	Medication: vitamin A	Improvement	NR
15	19	Btaiche 2011 [42]	BPD with DS	9	Copper	Neuropathy	Medication: copper	Improvement	Misdiagnosis
16	20	Chacko 2012 [43]	Gastric band	3	Vitamin B1; folate	Reduced vision	Medications: vitamin B12, folic acid, vitamin B	Permanent blindness	No post‐operative supplementation Poor oral nutrition Vomiting
17	21	Chami 2022 [44]	Gastric bypass	19	Copper	Bilateral upper and lower extremity weakness, numbness, and paraesthesia	Medication: copper	Improvement	NR
18	22	Chizooma 2023 [45]	RYGB	11	Zinc; selenium	Falls, weakness and dehisced surgical wound	Medication: nutritional supplementation including zinc and selenium, antibiotics. Surgical: Laparotomy and closure of wound	Improvement	Nonadherent to supplementation
19	23	Choi 2010 [46]	RYGB	6	Copper	Neuropathy	Medication: copper	Resolution	NR
20	24	Collazo‐Clavell 2004 [47]	RYGB	7	Vitamin D	Osteomalacia	Medication: calcium phosphate, ergocalciferol Other: stop bisphosphonates	Improvement	Misdiagnosis
21	25	Corbeels [48]	BPD	2	Vitamin D, Calcium, Phosphorus	Collapse; multiple vertebral fractures; steatorrhea	Medication: IM cholecalciferol; lipase, amylase and protease for pancreatic exocrine insufficiency	Improvement	NR
22	26	Di Stefani 2007 [49]	BPD	2	Vitamin A; Vitamin E	Dermatological complains	Medication: vitamin A, vitamin E, vitamin B; vitamin C; essential fatty acids, keratolytic ointment	Resolution	NR
23	27	Dixit 2023 [50]	RYGB	> 10	Vitamins A, B1, B6, C and E as well as zinc, copper	Generalised weakness; diffuse, pruritic and painful desquamating rash; weight loss, nausea, abdominal pain, diarrhoea, loss of taste, arthralgias and chills	Medication: antibiotics; zinc; copper, vitamin B9, vitamin B1, vitamin B6, vitamin C, vitamin D3, vitamin A, vitamin E, vitamin D2 capsule multivitamin tablet. Zinc oxide 40% paste and vitamin A cream applied. Protein supplements. Pancrelipase.	Cardiac arrest and death	Unclear
24	28	Eerdekens 2010 [51]	Gastric band	2	Vitamin K	Emergency C‐section and baby with intracranial haemorrhages	Medication for mother: TPN Surgery for mother: two procedures to relive obstruction from malpositioned band Interventions for baby: intubated, ventilation, ITU support Medications for baby: vitamin K, fresh frozen plasma FFP	Death of baby	No dietary counselling
25	29	Faiz 2007 [52]	Gastric bypass	9	Vitamin D	Musculoskeletal complaints	Medications: ergocalciferol	Improvement	Misdiagnosed
26	30	Fok 2012 [53]	RYGB	3	Vitamin A	Reduced vision	Medications: vitamin A, multivitamins	Improvement	Nonadherent to supplementation
26	31	Fok 2012 [53]	Jejuno‐ileal bypass	18	Vitamin A	Night blindness, eye discomfort	Medications: vitamin A	Resolution	Nonadherent to supplementation
27	32	Freitas 2019 [54]	RYGB	6	Vitamin B6; zinc	Skin rash	Medications: multivitamin, thiamine, folate, zinc, vitamin B6	Improvement before death of unknown cause	Alcohol excess Poor oral nutrition; Nonadherent to supplementation
28	33	Giannaccare 2020 [55]	BPD with DS	15	Vitamin A	Right corneal ulcer	Medication: vitamin A ointment, vitamin A Surgery: conjunctival flap	Monocular vision loss	Nonadherent to supplementation
28	34	Giannaccare 2020 [55]	BPD with DS	14	Vitamin A	Eye pain and light sensitivity	Medication: vitamin A ointment, vitamin A Intervention: Botox into levator superioris	Poor unilateral vision	Nonadherent to supplementation Financial difficulties
29	35	Govero 2022 [56]	RYGB	10	Phosphate, vitamin D, potassium, calcium	Progressive weakness, myalgia, exertional dyspnoea, peripheral neuropathy	Medication: potassium phosphate, calcitriol, ergocalciferol	Improvement	NR
30	36	Griffith 2009 [57]	RYGB	21	Copper	Peripheral neuropathy	Medication: copper, vitamin B6	Improvement	Nonadherent to supplementation
31	37	Haria 2005 [58]	Jejuno‐ileal bypass	32	Vitamin D	Osteomalacia (incidental finding)	Medication: vitamin D, calcium carbonate	Improvement before death of c/diff colitis, sepsis, GI haemorrhage, SVT 10 months after	NR
32	38	Herrera‐Martinez 2021 [59]	BPD	12	Zinc; Vitamin D; Copper; Vitamin A; Vitamin E	Lower extremity pitting oedema, glossitis, angular cheilitis, extensive plaques with erythema and desquamation, diffuse alopecia	Medication: zinc, oral pancrelipase supplements, vitamin A, vitamin E, calcifediol, copper, multivitamin with minerals and trace elements, thiamine, rifaximin Other: bland diet and protein supplementation	Resolution	Loss to follow up 7 years post‐procedure; not receiving vitamin supplements due to economic difficulties and work‐related problems
33	39	Huerta 2002 [60]	BPD	13	Vitamin A	Fatigue, oedema, dyspnoea during and after pregnancy	Medications: thiamine, folic acid, vitamin D, vitamin B6, vitamin E, ferrous sulfate, vitamin A Interventions: transfusion 2 units packed red blood cells	Resolution in mother Improvement in baby	LTFU with Nonadherent to supplementation
34	40	Joshi 2019 [61]	Jejuno‐ileal bypass	40	Copper	Neuropathy	Medications: copper Other: transfer to acute rehab hospital	Improvement Had bariatric surgery reversed	NR
35	41	Khambatta 2010 [62]	RYGB	16	Copper	Peripheral neuropathy	Medication: copper, multivitamins	Improvement	Nonadherent to supplementation
36	42	Kirkland 2022 [63]	RYGB	21	Copper	Peripheral neuropathy	Medication: copper, cyanocobalamin, multivitamin	Improvement	Nonadherent to supplementation
37	43	Larkin 2022 [64]	RYGB	20	Folate, vitamin D	Shortness of breath, fatigue, diarrhoea, aphthous ulcers, alopecia	Medication: prednisone, calciferol, folate, B12 and thiamine	Improvement	Nonadherent to supplementation Chronic alcohol use
38	44	Lee 2005 [65]	BPD with DS	3	Vitamin A	Reduced vision, night blindness, eye discomfort	Medication: multivitamins, artificial tears, topical erythromycin Intervention: punctal plugs inserted	Improvement	Nonadherent to supplementation
39	45	Lemieux 2019 [66]	BPD	26	Vitamin A	Monocular blindness, night blindness	Medication: vitamin A	Blindness	NR
40	46	Levanti 2022 [67]	BPD	14	Thiamine	Vomiting and worsening neurological signs	Medication: parenteral electrolyte solutions, thiamine replacement	Improvement	Reduced nutritional intake, delayed diagnosis
41	47	Lima de Carvalho 2022 [68]	DS	13	Vitamin A	Progressive Night blindness	Medication: vitamin A	Improvement	Nonadherent to supplementation and insufficient dose prescription
42	48	Mankaney 2014 [69]	RYGB	8	Zinc	Skin rash	Medication: zinc	Improvement	Nonadherent to supplementation Poor oral nutrition Financial difficulties
43	49	Martines 2018 [70]	Gastric band	2	Vitamin B1; Vitamin B12	Polyneuropathy	Medication: PEG for enteral nutrition	Improvement	LTFU
44	50	Merola 2012 [71]	RYGB	5	Vitamin B1	Wernicke's encephalopathy	Medication: thiamine, copper, zinc, folate, vitamin B12, vitamin D, multivitamins	Improvement	Alcohol excess Nonadherent to supplements
45	51	Monshi 2015 [72]	RYGB	4	Vitamin A	Phrynoderma (skin rash) whilst breastfeeding	Medication: high calorie parenteral nutrition, zinc chloride, sodium selenite, ferric chloride, vitamin A, vitamin D, vitamin E, vitamin C, vitamin B1, vitamin B2, vitamin B6, vitamin B12, folic acid, vitamin B5, vitamin H, nicotinamide, protein substitution, urea ointment, topical betamethasone dipropionate	Resolution	NR
46	52	Mouaffak 2014 [73]	RYGB	2	Folate	Resistant depression	Medication: supplementation according to local guidelines	Resolution	Nonadherent to supplementation Poor oral nutrition
47	53	Mouallem 2018 [74]	Partial gastrectomy with Roux‐en‐Y gastroenterostomy with BPD	6	Copper	Reduced vision and neurology	Medication: multivitamins, trace elements, selenium, vitamin D	Improvement	Nonadherent to supplementation
48	54	Naismith 2009 [75]	Gastric bypass	22	Copper	Optic neuropathy and myeloneuropathy	Medications: copper	Complete blindness	Nonadherent to supplementation
49	55	Papanastasiou 2020 [76]	RYGB	15	Vitamin D	Osteomalacia	Medications: iron, calcium gluconate, alpha‐calcidiol, calcium citrate Other: physiotherapy	Improvement	Nonadherent to supplementation
50	56	Pelizzo 2014 [77]	RYGB	5	Vitamin B12	Neural tube defect	Medication in mother: nutrient supplement during pregnancy Surgery in baby: closure of myelo‐meningocele on day 1	Further surgery required for baby: ventriculo‐peritoneal drainage tube positioned after 1 m	No nutritional evaluation postoperatively or prenatally
51	57	Pereira‐Cunill [78]	Gastroileal bypass	2	Vitamin A, Vitamin D, iron	Repeated episodes of hypoglycaemia	Medication: dietary modification, calcium and vitamin D supplements, rifaximin	Did not improve	NR
51	58	Pereira‐Cunill [78]	Gastroileal bypass	2	Vitamins A & E	Dyspepsia	NR	NR	NR
52	59	Pineles 2010 [79]	Gastric bypass	> 20	Copper	Visual loss and neuropathy	Medications: B12, copper	Stabilisation	NR
53	60	Rana 2016 [80]	RYGB	13	Zinc	Acrodermatitis enteropathica (skin rash)	Medication: zinc TPN, micronutrients	Improvement	Poor oral nutrition LTFU
54	61	Rothkopf 2006 [81]	Jejunoileostomy	20	Vitamin A	Neuropathy	Medication: TPN, K+, Mg++, vitamin B12, vitamin A, vitamin D	Resolution	NR
54	62	Rothkopf 2006 [81]	Jejunoileostomy	35	Vitamin A	Neurology	Medication: TPN, vitamin A, vitamin E, vitamin B6, K+ and Mg++; vitamin B12, vitamin A	Resolution	NR
55	63	Santarpia 2020 [82]	BPD	13	Vitamin D	Osteomalacia and osteoporosis	Intervention: fracture stabilisation Other: physiotherapy	Resolution	LTFU
56	64	Shirodkar 2023 [83]	RYGB	15	Niacin, iron, zinc, potassium, magnesium	Decline in physical and cognitive function, rash, lower extremity pain and weakness, diarrhoea, vomiting	Medication: Iron, potassium, zinc, vitamin B complex, niacin	Improvement	Co‐morbidities (breast cancer, depression) Alcohol consumption
57	65	Sindaco 2020 [84]	BPD	17	Vitamin E; Vitamin A	Decreased vision and hearing	Medication: vitamin A, vitamin E	Improvement	Malabsorption syndrome
58	66	Smelt 2018 [85]	RYGB	2	Vitamin B12	Folic acid treatment with untreated vitamin B12 deficiency (neuropathy, visual disturbance, behaviour change)	Medication: vitamin B12 Other: stop folic acid	Resolution	Folic acid given stat but B12 to be given by GP in 3 weeks' time
59	67	Smets 2006 [86]	BPD	8	Vitamin A	Incidental deficiency found at week 16 pregnancy	Medication: multivitamins, vitamin B12, iron, folic acid, parenteral nutrition	Baby born with bilateral microphthalmia, simian crease, cerebral ventricular asymmetry	Previous 3 pregnancy did not result in live birth
60	68	Soares 2019 [87]	BPD	10	Vitamin E	Neuropathy	Medication: parenteral and enteral nutrition, iron, vitamins, pro‐biotics, pancreatic enzymes	Required reconversion surgery	LTFU Nonadherent to supplementation
61	69	Steenackers 2021 [88]	BPD	24	Vitamin A, vitamin E, zinc	Weakness, recurrent falls, confusion, unresponsive episodes, anorexia and weight loss	Medication: parenteral nutrition with additional micronutrient supplementation	Improvement	Medical comorbidities Nonadherent to supplementation
62	70	Stephens 2012 [89]	BPD	5	Vitamin D; Vitamin A	Neuropathy and night blindness	Medication: Calcichew (calcium and cholecalciferol), vitamin A, vitamin D, ferrous sulfate, vitamin B6, forceval	Improvement but found to have adrenal insufficiency	Nonadherent to supplementation
63	71	Stroh 2010 [90]	DS	4	Vitamin A	Loss of vision and night blindness	Medication: FrekaVit, vitamin A, calcium, vitamin D, Kabiven	Resolution	Nonadherent to supplementation
64	72	Tatineni 2020 [91]	Gastric bypass	14	Copper	Chest pain, shortness of breath and non‐specific symptoms for example, fatigue, abdominal pain, arthralgia	Medication: 3 units packed red blood cells, copper Other: stop zinc	Resolution after intensification of treatment	Zinc excess
65	73	Tsai 2017 [92]	Gastric bypass	7	Thiamine	Hepatomegaly, severe malnutrition, peripheral neuropathy, portal hypertension, varices, coagulopathy	Medication	Improvement	NR
66	74	Van Mieghem 2008 [93]	Gastric band	2	Vitamin K	Reflux and vomiting during pregnancy and inability to ingest food	Medication: fluids, vitamins, TPN Other: restricted oral foods Surgery: emergency C section for foetal tachycardia	Death of baby after intensive care withdrawn on day 7	Prolonged maternal vomiting with slippage of gastric band
67	75	Velasco 2009 [94]	Vertical banded gastroplasty	4	Thiamine	Wernicke's encephalopathy	Medication: thiamine BD, vitamin B complex	Resolution Eventually has revision surgery to BPD	Nonadherent To supplementation
68	76	Vick 2015 [95]	Gastric bypass	10	Zinc	Skin rash	Medications: zinc, copper, iron	Improvement	NR
69	77	Waserman 2015 [96]	RYGB	8	Vitamins A, D, E, copper, zinc, calcium	Major depressive episode	Medication: valproic acid, vitamin B12, lipase, amylase‐protease, copper, vitamin A, vitamin B 100 complex, vitamin D, vitamin E, zinc citrate, calcium citrate, psyllium powder, loperamide, acetaminophen, protein‐rich supplement, crossover of venlafaxine to mirtazapine	Improvement	NR
70	78	Wilson 2014 [97]	BPD	6	Vitamins A, D, E, copper, zinc, calcium	Osteoporosis, skin rash and reduced night vision	Surgery: open reduction and internal fixation Medication: vitamin A, vitamin D, trace element	Improvement of night blindness 6 months later died on ITU after wound dehiscence, sepsis and encephalopathy	LTFU
71	79	Yahalom 2019 [98]	SG	5	Zinc	Dyspnoea and heart failure	Medication: furosemide, carvedilol, enalapril, zinc	Resolution	NR
72	80	Yarandi 2014 [99]	RYGB	13	Copper	Neuropathy and visual loss	Medication: copper sulfate, copper gluconate, vitamin B6, thiamine, vitamin D	Improvement	Use of zinc denture cream
73	81	Yu 2019 [100]	RYGB	12	Copper	Acute on chronic liver failure	Medication: copper	Improvement before death from fungal sepsis and multiorgan failure	NR
74	82	Zouridaki 2014 [101]	RYGB	8	Vitamin B12	Infected abscess	Medication: vitamin B12, amikacin	Improvement	LTFU
74	83	Zouridaki 2014 [101]	RYGB	2	Vitamin B12	Skin rash and ulcer	Medication: vitamin B12, calcium, vitamin D	Improvement	NR

Abbreviations: BPD, biliopancreatic diversion; DS, duodenal switch; LTFU, lost to follow up; NR, not reported; RYGB, Roux‐en‐Y gastric bypass; SG, sleeve gastrectomy.

Fourty‐one cases (reported in 37 articles) were from the USA [30, 31, 32, 33, 35, 39, 41, 42, 43, 44, 46, 47, 50, 52, 54, 56, 57, 58, 60, 61, 62, 63, 64, 65, 66, 68, 69, 71, 75, 79, 80, 81, 83, 91, 95, 99, 100], 9 from Italy (8 articles) [40, 49, 55, 67, 70, 77, 82, 84], 6 from Belgium [36, 48, 51, 86, 88, 93], 5 from Spain (4 articles) [34, 59, 78, 94], 4 from the UK [28, 45, 89, 97], 3 from France (2 articles) [38, 73], 3 from Greece (2 articles) [76, 101], 2 from each of Australia (1 article) [53], Germany [29, 90] and Israel [74, 98] and one from each of Austria [72], Brazil [87], Canada [96], Denmark [37], Taiwan [92] and Netherlands [85]. Studies were published between 2002 and 2023.

Five included articles were case series [30, 38, 51, 92, 100] and the remainder were case reports.

Characteristics of included cases are summarised in Table 1.

### 3.2 Quality Assessment

Quality assessment results are reported in Table 2 for case reports and case series in Table 3. Studies were of variable quality, with none meeting all the quality assessment criteria.

**TABLE 2:** Critical appraisal of case reports.

1st author and year	Demographics clearly described?	History clearly described?	Clinical condition on presentation clearly described?	Diagnostic tests clearly described?	Interventions clearly described?	Post‐intervention clinical condition clearly described?	Takeaway lessons?
AlHassany 2014 [28]	Unclear	Yes	Yes	No	No	Yes	Yes
Alkurdi 2021 [29]	Unclear	Yes	Yes	Yes	Unclear	Yes	Yes
Amin 2022 [31]	Unclear	Unclear	Yes	Yes	Yes	Yes	Yes
Atreja 2003 [32]	Unclear	Unclear	Yes	Yes	No	No	Yes
Bae‐Harboe 2012 [33]	Unclear	Unclear	Yes	Yes	Unclear	Unclear	Yes
Barro 2017 [34]	No	Unclear	Yes	Yes	Yes	Yes	Yes
Beh 2013 [35]	Unclear	Yes	Yes	Yes	No	Yes	Yes
Benhalima 2009 [36]	Unclear	Unclear	Yes	Yes	Yes	Unclear	Yes
Bergmann 2020 [37]	Unclear	Unclear	Yes	Yes	No	Unclear	Yes
Bowley 2017 [39]	Unclear	Yes	Yes	Yes	Yes	Yes	No
Brancatella 2017 [40]	Unclear	Unclear	Yes	Yes	Yes	Yes	Yes
Breazzano 2023 [41] (case ID 17)	Unclear	Unclear	Unclear	Unclear	Yes	Yes	Yes
Breazzano 2023 [41] (case ID 18)	Unclear	Unclear	Unclear	Unclear	Yes	Unclear	Yes
Btaiche 2011 [42]	Unclear	Unclear	Yes	Yes	Yes	Yes	Yes
Chacko 2012 [43]	Unclear	Yes	Yes	Yes	No	Unclear	Yes
Chami 2022 [44]	Unclear	Unclear	Yes	Yes	Yes	Yes	Yes
Chizooma 2023 [45]	Unclear	Unclear	Unclear	Yes	Yes	Unclear	Yes
Choi 2010 [46]	Yes	Unclear	Unclear	Yes	Yes	Yes	Yes
Collazo‐Clavell 2004 [47]	Unclear	Unclear	Unclear	Yes	Yes	Yes	Yes
Corbeels [48]	Unclear	Unclear	No	Yes	Unclear	No	Yes
Di Stefani 2007 [49]	Unclear	Unclear	Yes	Yes	No	No	Yes
Dixit 2023 [50]	Unclear	Unclear	Yes	Yes	Yes	Yes	Yes
Faiz 2007 [52]	Unclear	Unclear	No	No	Yes	Unclear	Yes
Fok 2012 [53] (case ID 30)	Unclear	Unclear	Unclear	Yes	Yes	Yes	Yes
Fok 2012 [53] (case ID 31)	Unclear	Unclear	No	Unclear	Yes	Unclear	Yes
Freitas 2019 [54]	Unclear	Yes	Yes	Yes	Yes	Unclear	Yes
Giannaccare 2020 [55] (case ID 33)	Unclear	Unclear	Unclear	Yes	Yes	Unclear	Yes
Giannaccare 2020 [55] (case ID 34)	Unclear	Unclear	Unclear	Yes	Yes	Unclear	Yes
Govero 2022 [56]	Unclear	Unclear	Yes	Yes	Yes	Unclear	Unclear
Griffith 2009 [57]	Yes	Unclear	Yes	Yes	Yes	Yes	Yes
Haria 2005 [58]	Yes	Yes	Unclear	Yes	Yes	Yes	Yes
Herrera‐Martinez 2021 [59]	Yes	Unclear	Yes	Yes	Yes	Yes	Yes
Huerta 2002 [60]	Unclear	Unclear	Yes	Yes	Unclear	Unclear	Yes
Joshi 2019 [61]	Unclear	Unclear	Yes	Yes	Unclear	Unclear	Yes
Khambatta 2010 [62]	Yes	Unclear	Yes	Yes	Yes	Unclear	Yes
Kirkland 2022 [63]	Unclear	Unclear	Yes	Yes	Yes	Yes	Yes
Larkin 2022 [64]	Unclear	Unclear	Unclear	Yes	Yes	Yes	Yes
Lee 2005 [65]	Unclear	Unclear	Unclear	Yes	Yes	Yes	Yes
Lemieux 2019 [66]	Unclear	No	No	No	Yes	Unclear	Yes
Levanti 2022 [67]	Unclear	Unclear	Yes	Yes	Yes	Yes	Yes
Lima de Carvalho 2022 [68]	Unclear	Unclear	Unclear	Yes	Yes	Yes	Yes
Mankaney 2014 [69]	No	No	Unclear	Unclear	No	Unclear	No
Martines 2018 [70]	Unclear	Unclear	No	No	No	Unclear	Yes
Merola 2012 [71]	Unclear	Yes	Yes	Yes	Yes	Unclear	Yes
Monshi 2015 [72]	Unclear	Unclear	Yes	Yes	Yes	Yes	Yes
Mouaffak 2014 [73]	Unclear	Unclear	No	Unclear	No	Unclear	Yes
Mouallem 2018 [74]	Unclear	Unclear	Yes	Yes	No	Unclear	Yes
Naismith 2009 [75]	Unclear	Yes	Yes	Yes	No	Yes	Yes
Papanastasiou 2020 [76]	Unclear	Unclear	Unclear	Yes	Unclear	Yes	Yes
Pelizzo 2014 [77]	Unclear	Unclear	Unclear	Unclear	No	No	Yes
Pereira‐Cunill [78] (case ID 58)	Unclear	No	No	Yes	Unclear	Unclear	Yes
Pereira‐Cunill [78] (case ID 59)	Unclear	No	No	Yes	No	No	Yes
Pineles 2010 [79]	Unclear	Yes	Yes	Yes	No	Unclear	Yes
Rana 2016 [80]	Unclear	Unclear	Unclear	Yes	Yes	Unclear	Yes
Rothkopf 2006 [81] (case ID 61)	Unclear	Unclear	Yes	Yes	Yes	Unclear	Yes
Rothkopf 2006 [81] (case ID 62)	Unclear	Unclear	Yes	Yes	Yes	Yes	Yes
Santarpia 2020 [82]	Unclear	Unclear	Unclear	Yes	No	No	Yes
Shirodkar 2023 [83]	Unclear	Yes	Yes	Yes	Yes	Yes	Yes
Sindaco 2020 [84]	Unclear	Unclear	Unclear	Yes	Yes	Yes	Yes
Smelt 2018 [85]	Unclear	Unclear	Yes	Yes	Yes	Yes	Yes
Smets 2006 [86]	Unclear	Unclear	Unclear	No	No	Yes	Yes
Soares 2019 [87]	Unclear	Unclear	Unclear	Yes	Unclear	Yes	Yes
Steenackers 2021 [88]	Unclear	Unclear	Yes	Yes	Yes	Yes	Yes
Stephens 2012 [89]	Unclear	Unclear	Unclear	Yes	Yes	Yes	Yes
Stroh 2010 [90]	Unclear	Yes	Yes	Unclear	Yes	Unclear	Yes
Tatineni 2020 [91]	Unclear	Yes	Yes	Yes	Yes	Yes	Yes
Van Mieghem 2008 [93]	Unclear	Unclear	Unclear	Yes	Unclear	Yes	Yes
Velasco 2009 [94]	Unclear	Unclear	Unclear	Yes	Yes	Yes	Yes
Vick 2015 [95]	Yes	Unclear	Yes	Yes	Unclear	Unclear	Yes
Waserman 2015 [96]	Unclear	Yes	Unclear	Yes	Yes	Unclear	Yes
Wilson 2014 [97]	Unclear	Unclear	Unclear	Yes	Unclear	Yes	Yes
Yahalom 2019 [98]	Unclear	Unclear	No	Unclear	Unclear	Yes	Yes
Yarandi 2014 [99]	Unclear	Unclear	Unclear	Yes	Yes	Yes	Yes
Zouridaki 2014 [101] (case ID 82)	Unclear	Unclear	Unclear	Yes	Yes	Unclear	Yes
Zouridaki 2014 [101] (case ID 83)	Unclear	Unclear	Unclear	Yes	Unclear	Unclear	Yes

**TABLE 3:** Critical appraisal of case series.

1st author and year	Clear inclusion criteria?	Condition measured in a standard, reliable way?	Valid methods used for participant identification?	Consecutive inclusion of participants?	Complete inclusion of participants?	Clear reporting of demographics?	Clear reporting of clinical information?	Outcomes of follow up results of cases clearly reported?	Clear reporting of presenting sites?	Appropriate statistical analysis
Al‐Shoha 2009 [30]	Unclear	Yes	Yes	No	No	Yes	Yes	Yes	No	N/A
Bétry [38]	Yes	Yes	Yes	Yes	No	Unclear	No	No	No	N/A
Eerdekens 2010 [51]	No	No	Yes	No	No	Unclear	Yes	Unclear	No	N/A
Tsai 2017 [92]	Yes	Yes	Yes	Yes	Yes	Yes	No	No	No	N/A
Yu 2019 [100]	No	Unclear	Yes	No	No	Unclear	Yes	Yes	No	N/A

### 3.3 Participants

70 (84%) cases were female and 13 were male. Age ranged from 22 to 74. Four case reports included pregnant women [51, 77, 86, 93], and three post‐partum women [60, 72, 98], one of which focused on breastfeeding women [72].

### 3.4 Procedures

Bariatric procedures were categorised according to how they were reported in the published articles.

#### 3.4.1 Types of Bariatric Procedures

Thirty‐one cases (reported in 29 articles) had a Roux‐en‐Y gastric bypass (RYGB) [30, 31, 34, 39, 45, 46, 47, 50, 53, 54, 56, 57, 62, 63, 64, 69, 71, 72, 73, 76, 77, 80, 83, 85, 92, 96, 99, 100, 101]. Eighteen cases had BPD [30, 36, 40, 41, 48, 49, 59, 60, 66, 67, 74, 82, 84, 86, 87, 88, 89, 97]. Eight cases reported on “gastric bypass” surgery with no further details of the specific procedure provided [33, 37, 44, 52, 75, 79, 91, 95]. Six cases had a laparoscopic gastric band [35, 38, 43, 51, 70, 93]. Six cases (reported in five articles) had BPD with duodenal switch [32, 41, 42, 55, 65]. Two cases were described as “duodenal switch” with no further details [68, 90]. Six cases (in five articles) had jejunoileal bypass [29, 53, 58, 61, 81]. Two cases (in one article) reported on sleeve gastrectomy [98]. One case reported on each of the following procedures: one anastomosis gastric bypass [38]; vertical banded gastroplasty [94]; gastroileal bypass [78]; and sleeve gastrectomy and duodenal switch [28].

The range of time since surgery was 2 to 40 years (median 8.5, IQR 11), excluding three case reports in which the time since surgery given was only reported as > 3 years [38], > 10 years [50] and > 20 years [79].

### 3.5 Deficiencies

Patients in 65 cases had a ‘main’ deficiency, which related to the presenting complaint and was the primary focus of the report, along with other reported incidental or long‐standing deficiencies. The remaining cases (*n* = 18) reported multiple deficiencies. The reports including pregnant women [51, 60, 77, 86, 93] and breastfeeding women [72] are discussed both under the relevant deficiency, and in a separate section on pregnancy and breastfeeding.

#### 3.5.1 Vitamin A

Fifteen cases (in 11 articles) reported primarily on vitamin A deficiency [41, 53, 55, 60, 65, 66, 68, 72, 81, 86, 90].

Eleven cases (in eight articles) presented with primarily ophthalmological symptoms [41, 53, 55, 60, 65, 66, 68, 90]. Of these, eight cases (in seven articles) presented with night blindness [41, 53, 60, 65, 66, 68, 90], one with visual deterioration [53], one with a corneal ulcer [55], and one with eye pain and sensitivity [55].

Of those not presenting with ophthalmological symptoms, a single case presented with phrynoderma with no night blindness whilst breastfeeding [72]; one was identified before any symptoms developed through monitoring during pregnancy due to a history of multiple pregnancy complications and miscarriages [86]; one with vertigo, confusion, dysarthria, and ataxia [81]; and one case presented with slurred speech, paraesthesia, and muscle weakness with no night blindness [81].

All cases received vitamin A replacement. The routes of administration varied. None of the cases received oral vitamin A only, but three cases reported receiving oral vitamin A initially followed by another route of replacement [41, 66, 86]. Eight cases (reported in six articles) received intramuscular (IM) replacement [41, 53, 55, 60, 66, 68] and five cases (in four articles) received intravenous (IV) replacement via total parenteral nutrition (TPN) [72, 81, 86, 90]. Two cases (in one article) received vitamin A eye ointment [55]. One case received vitamin A as part of multivitamin supplementation, but the route was unclear [65].

Supplementation of other additional nutrients or multivitamins was reported in eight cases (seven articles) [53, 60, 65, 72, 81, 86, 90].

Ocular surgery (Gunderson conjunctival flap) was required in one case [55]. Other ocular treatments included botox into levator superioris [55]; bilateral lower punctal plugs [65], artificial tears [65] and topical erythromycin [65].

Non‐ocular treatments included topical 10% urea ointment and 30% beclometasone dipropionate for phrynoderma [72] and red blood cell transfusion [60].

Treatment generally improved, if not resolved, clinical and biochemical derangements. In three cases (in two articles), however, monocular visual loss persisted and was permanent [55, 66].

#### 3.5.2 Copper

Fifteen cases (in 15 articles) reported on copper deficiency [31, 39, 42, 44, 46, 57, 61, 62, 63, 74, 75, 79, 91, 99, 100]. Eight cases reported concurrent vitamin deficiencies including zinc [42, 100], iron [42, 79, 91, 99], vitamin A [42, 74], vitamin E [42], vitamin D [42, 57, 74, 99], vitamin B6 [57, 99], vitamin B1 [74, 99] and vitamin B12 [63, 79].

Presentations were predominantly neurological (*n* = 10) [31, 39, 42, 44, 46, 57, 61, 62, 63, 91] or neurological and ophthalmological together (*n* = 4) [74, 75, 79, 99]. One of the patients with ophthalmological symptoms also had a vitamin A deficiency [74]. One case presented with acute on chronic liver failure [100].

Neurological signs and symptoms included neuropathic pain, paraesthesia, sensory loss, weakness, ataxia, gait disturbance, urinary retention, and falls. Ophthalmological signs and symptoms included loss of vision and blurred vision. Fatigue, confusion, and chest pain were additional features of several patients' presentations.

All patients had supplementary copper: one received IV, IM and oral copper [63], six received IV and oral copper [39, 42, 61, 62, 75, 99], two received IV copper alone [31, 57] and four oral replacement alone [44, 46, 79, 100]. One case did not report the route of copper supplementation [91]. One case was treated with “vitamins and trace elements”, but the report did not explicitly state whether this included copper [74].

Other management (each mentioned once) included: pregabalin and gabapentin [39], packed red blood cells [91], stopping zinc supplementation [91] and transfer to a rehabilitation hospital [61]. Six patients received some combination of other vitamins and mineral supplementation in addition to copper [57, 62, 63, 74, 79, 99]. One patient went on to have their bariatric surgery reversed [61].

In most cases, treatment led to improvements in symptoms and/or normalisation of test results [31, 39, 42, 44, 46, 57, 61, 62, 63, 75, 91, 99]. In several cases, residual symptoms remained, including persistent paresthesia [44, 62, 63], lower limb weakness [44], reduced mobility [57, 61] and blindness [75]. In one case, treatment led to a halt in deterioration but not to improvement [79]. One case reported a “dramatic improvement”, with only minimal residual muscle weakness and pericardial effusion [74]. One case initially improved but subsequently died from fungal sepsis and multiorgan failure [100].

### 3.6 Vitamin D

Twelve cases (in 10 articles) reported primarily on vitamin D deficiency [30, 32, 34, 36, 40, 47, 52, 58, 76, 82]. Nine of these cases also had associated hypocalcaemia [30, 32, 34, 40, 47, 52, 58, 76, 82]. Five cases reported other nutritional deficiencies, including vitamin A [32, 34, 40, 82], vitamin B12 [32, 34, 76], zinc [32], vitamin E [82], magnesium [34] and iron [76].

Ten cases (in eight articles) presented with musculoskeletal symptoms, including joint and bone pain, muscle weakness, and decreased mobility [30, 32, 34, 36, 47, 52, 76, 82]. One of these was found to have non‐traumatic fractures [34]. Two were identified incidentally, one after presenting with a sub‐occlusive bowel episode [40] and the other after admission for lymphoedema with cellulitis and an ulcer [58]. Nine cases (in seven articles) had osteomalacia [30, 32, 34, 40, 58, 76, 82]. Two had osteoporosis [47, 82]. One case had a Brown tumour of the bone from secondary hyperparathyroidism [36]. All but one case [82] was reported as receiving vitamin D supplementation, via many different formulations, doses, and routes. All but two of the cases [52, 82] were reported as receiving supplemental calcium, and the two cases that did not were both recorded as being calcium deficient.

One case required surgical management of fractures [34], one case Brown tumour removal [36] and one case fracture fixation and physiotherapy [82]. Stopping bisphosphonates was part of the management of one patient [47]. Three cases received additional nutrient supplementation [32, 34, 76].

Management led to initial improvement in clinical condition in all cases (improvement in symptoms and/or normalisation of test results), but one patient died 10 months after presentation due to *C. difficile* colitis, septicaemia, gastrointestinal haemorrhage, and supraventricular tachycardia [58].

### 3.7 Zinc

Six cases (in six articles) reported primarily on zinc deficiency [33], [59], [69, 80], [95, 98]. Three cases also mentioned other deficiencies including copper [59, 95] vitamin D [59], vitamin A [59], vitamin E [59] and vitamin B6 [80].

Five presented with dermatological conditions including painful, pruritic, scaly, and erythematous rashes, desquamation, angular cheilitis, and alopecia [33, 59, 69, 80, 95]. One presentation included lower extremity pitting oedema [59], one oedematous palms and soles [33], and one case presented with dyspnoea and signs of heart failure [98].

All cases received zinc supplementation, two IV [59, 95], one IV followed by oral [80], one oral [98] and two route unclear [33, 69]. Two received some combination of other vitamins and mineral supplementation in addition to zinc [59, 95]. One case received TPN [80]. One case was treated with a simple diet, a high protein supplement, and rifaximin [59]. The case presenting with heart failure symptoms was given furosemide, carvedilol, and enalapril in addition to zinc supplementation [98].

Treatment led to rapid and complete or near complete resolution in all six cases.

### 3.8 Vitamin B12 and Folate

Three case reports (in two articles) focused on vitamin B12 deficiency [77, 101], two on folate deficiency [64, 73] and one on vitamin B12 and folate deficiency together [85]. Co‐occurring vitamin deficiencies included vitamin D [77, 85, 101], vitamin A [77], iron [85] and zinc [101].

Presenting complaints were diverse. One case presented with each of: A large abscess [101]; recurrent erythematous and pustular skin eruptions leading to ulcers [101]; shortness of breath and fatigue [64]; and treatment‐resistant depression [73]. The case with both vitamin B12 and folate deficiency presented with tinnitus, palpitations, blurred vision and neurological symptoms after treatment with folate before the concurrent vitamin B12 deficiency was identified [85]. Four cases received vitamin B12 replacement [64, 85, 101], three of which reported the route was IM and the fourth unspecified. One case received only oral folate supplementation [73]. The two cases with dermatological presentations received antibiotics [101]. All cases improved at follow‐up, with two reporting complete resolution [85, 101].

### 3.9 Thiamine

Five cases (in five articles) focused on thiamine deficiency [35, 71], [67, 92, 94].

Three presented with Wernicke's encephalopathy [35, 71, 94]. One presented with recurrent vomiting and worsening neurological signs [67] and one with hepatomegaly, portal hypertension, varices, coagulopathy and peripheral neuropathy [92]. Three cases received IV thiamine [35, 67, 71], one IM thiamine [94] and one “medical treatment” [92]. One case had subsequent revisional surgery [94]. All improved following treatment.

### 3.10 Selenium

No studies focused on selenium alone as the main deficiency. Seven cases (in six articles) discussed selenium deficiency, one as a focus of the article alongside zinc deficiency [45], the others as one of a number of deficiencies, but not the main deficiency discussed [32, 38, 80, 96, 97].

### 3.11 Other Deficiencies

In two cases the main deficiency discussed was vitamin K [51, 93]. In both cases the deficiency was identified during pregnancy, and in both cases the baby died.

In two cases the main deficiency was vitamin E [29, 87]. Both received parenteral and enteral nutrition with vitamin supplementation leading to improvement. One required surgical revision [87].

One case focused on vitamin B3 (niacin) deficiency [83], presenting with a decline in physical and cognitive function against a background of breast cancer, depression and increased alcohol consumption. Symptoms improved with niacin supplementation. One case focused on iron deficiency [37] presenting with tiredness and dyspnoea, which improved with oral and IV iron.

### 3.12 Multiple Deficiencies

Eighteen cases (in 15 articles) did not discuss a “main deficiency” but multiple deficiencies, as detailed in Table 1, [28, 38, 43, 45, 48, 49, 50, 54, 56, 70, 78, 84, 88, 89, 96, 97].

Presentations were broad and often affected multiple body systems. They included neurological symptoms [28, 38, 56, 70, 89], gastrointestinal symptoms [28, 38, 48, 50, 78], visual symptoms [28, 43, 84, 89] and dermatological symptoms [49, 50, 89]. Many cases also presented with non‐specific symptoms such as generalised weakness [45, 50, 56, 88, 89], collapse/falls [45, 48, 70, 88], fatigue [56] and myalgia [56]. One case presented with each of poor wound healing [45], non‐traumatic fractures [48] and amenorrhoea [89]. Eleven cases were treated with vitamin supplementation [28, 43, 45, 48, 49, 50, 56, 78, 84, 88, 89] and four cases (in three articles) with artificial nutrition [38, 70, 88]. Eight cases improved with treatment [45, 48, 49, 56, 70, 84, 88, 89], in three cases (in two articles) the outcome was not reported [38, 78], in one case with vomiting and abdominal discomfort did not improve [78], one case had residual night blindness [28], one was legally blind [43] and one case died from cardiac arrest related to pulmonary oedema [50].

### 3.13 Pregnancy and Post‐Partum

Four case reports included pregnant women [51, 77, 86, 93], and three post‐partum women [60, 72, 98], one of which focused on breastfeeding women [72].

Three of the case reports focused on vitamin A deficiency [60, 72, 86], two on vitamin K [51, 93], one on vitamin B12 [77] and one on zinc [98]. Other vitamin deficiencies discussed, but not the focus of the cases, were vitamin A [51, 77, 93] vitamin D [60, 72, 77], vitamin B12 [86], vitamin E [60, 72], calcium [60], and zinc [60, 72].

Among the pregnant cases, two presented with persistent vomiting [51, 93]. One was identified incidentally on routine bloods [86]. In two cases, problems with the foetus or baby preceded the identification of the mother's nutritional deficiency [51, 77]. One of each of the post‐partum cases presented with: phrynoderma [72], dyspnoea and signs of heart failure [98]; and fatigue, light‐headedness and night blindness [60].

In two cases the baby died [51, 93]. In two cases the baby had an adverse outcome (myelomeningocele [77], microphthalmia, simian crease and cerebral ventricular asymmetry [86]). In one case lasting retinal damage could not be excluded [60].

### 3.14 Contributing Factors

Factors reported as contributing to the development of late nutritional deficiencies after bariatric surgery were extracted where reported. Fifty‐one cases (in 48 articles) included contributing factors [28, 29, 30, 31, 32, 34, 36, 40, 41, 42, 43, 45, 47, 51, 52, 53, 54, 55, 57, 59, 60, 62, 63, 64, 65, 67, 68, 69, 70, 71, 73, 74, 75, 76, 77, 80, 82, 83, 86, 87, 88, 89, 90, 94, 96, 97, 101]. These were divided into patient factors and healthcare factors.

#### 3.14.1 Patient Factors

Twenty‐one cases (20 articles) reported patient non‐adherence to vitamin supplementation [45, 53, 54, 55, 60, 63, 64, 65, 68, 69, 70, 71, 73, 74, 76, 87, 88, 89, 90, 96]. Three cases highlighted financial difficulties as a contributing factor to inadequate vitamin supplementation [55, 59, 69]. Perceived lack of efficacy of supplementation was reported in one case [55].

A further 16 cases (16 articles) reported insufficient vitamin supplementation as a contributing factor but did not report further details on the cause of insufficient supplementation (e.g., prescribing, adherence) [29, 30, 36, 40, 41, 43, 53, 57, 59, 60, 62, 75, 77, 80, 94, 101].

A nutritionally unbalanced diet was discussed in eight cases [40, 43, 54, 64, 67, 69, 73, 80]. In five cases, excessive alcohol intake was a contributing factor [35, 54, 64, 71, 83].

In several cases uncontrolled symptoms were highlighted as contributing factors. Four cases mentioned vomiting [35, 43, 67, 93], one diarrhoea [28] and one pain [43].

In three cases, excessive zinc exposure led to copper deficiency (in the form of zinc denture cream [39, 99] or excess non‐prescribed supplementation [91]). Three cases discussed comorbidities as contributing factors. Two reported depression [71, 83], one breast cancer [83] and one herpes encephalitis [88].

#### 3.14.2 Healthcare Factors

In 14 cases (12 articles) the diagnosis of vitamin deficiency was delayed [28, 30, 31, 40, 42, 47, 52, 60, 67, 75, 82, 86]. In 11 of these 14 cases (9 articles) this was due to initial misdiagnosis [30, 31, 42, 47, 52, 60, 67, 75, 82] and in 4 of these 14 cases (2 articles) an initial misdiagnosis led to inappropriate treatment [30, 47]. In one case, delayed test results were a contributing factor [31].

Loss of the patient to follow‐up after bariatric surgery was reported in nine cases [34, 36, 40, 59, 60, 82, 87, 89, 96] and inadequate follow‐up in three cases [80, 97, 101].

Two cases mentioned the role of primary care. One case explicitly stated that a contributing factor was loss to follow up with the General Practitioner [96] and another highlighted misdiagnosis in Primary Care [32].

Some articles highlighted that once the first nutritional deficiency was diagnosed, others were looked for and found [32] and when this was not done, it led to adverse outcomes [60].

Lack of health professional knowledge about adequate supplement doses was mentioned in three cases (one article) [30].

Insufficient patient education was identified in two cases [51, 63]. One case reported lack of input from a nutritionist post‐operatively as a contributing factor [77].

## 4 Discussion

This is the first systematic review of case reports and case series describing micronutrient deficiencies occurring at least 2 years following bariatric surgery. Although case reports are considered lower down the evidence hierarchy [102], their synthesis provides details on the sequelae for real patients of micronutrient deficiencies and highlights issues around follow‐up, contributing factors, correct recognition of deficiencies by clinicians, and variability in treatment.

The included cases reported a wide range of micronutrient deficiencies. It is not possible to comment on the incidence of deficiencies from case reports, but they do highlight challenges in identifying the less common micronutrient deficiencies and recognising the range of different presentations of late micronutrient deficiencies that can occur. A retrospective cohort study of 3137 participants with ≥ 3 years follow‐up post‐bariatric surgery in primary care in the UK found that the greatest proportions of patients with a record of annual nutritional blood tests were for tests routinely conducted in primary care such as haemoglobin (proportion having each of these routine tests varied between 44.9% (*n* = 629/1400) and 61.2% (*n* = 653/1067)) [11]. Annual proportions of blood tests specific to bariatric surgery were low. For example, copper measurement percentage varied between 1.2% (*n* = 10/818) and 1.5% (*n* = 16/1067) where recommended. Anaemia was the most commonly identified deficiency, and annual proportions of patients with prescriptions for recommended nutritional supplements were low. It is notable that despite the high prevalence of iron deficiency anaemia post‐bariatric surgery (estimated around 15% post‐malabsorptive bariatric procedures [103, 104]), only one case included in this review focused on iron deficiency anaemia, suggesting publication bias in favour of reports of more unusual nutritional deficiencies. Many included cases were of patients with BPD/BPD with DS, procedures which are less commonly performed now due to the high risk of nutritional complications, although clinicians will still see patients who had these procedures historically [88].

Some of the cases reported factors contributing to nutritional deficiencies, but these reports were generally a small part of the case reports and were not of sufficient richness to be amenable to more in‐depth qualitative analysis. Many reported a contributing factor, such as insufficient vitamin supplementation, but did not explore the causes behind this. Lawton et al. developed an evidence‐based framework of factors contributing to patient safety incidents in hospitals, which moves from active failures (errors, mistakes etc.) at the centre, radiating out to consider situational factors such as patient and team characteristics, working conditions, organisational factors, and the external policy context [105]. Future studies could explore the wider factors influencing immediate contributors to post‐bariatric surgery nutritional deficiencies.

According to a review by Lupoli et al. (2017) common problems caused by nutritional deficiencies post‐bariatric surgery include anaemia, osteoporosis, neurological disorders, and malnutrition [106]. The authors highlight that many people with obesity have nutritional deficiencies pre‐bariatric surgery, which may then be exacerbated following their procedure, so nutritional assessment, support, and follow‐up are important throughout their care.

A nationwide retrospective study in France (conducted 2013–2022, (*n* = 55 941)) comparing post‐bariatric surgery pregnancies with obesity‐ and comorbidity‐matched controls found reduced risks of gestational hypertension, pre‐eclampsia and gestational diabetes, but increased risks of small for gestational age (SGA) and prematurity, particularly if the pregnant person was malnourished, emphasising the need for optimal nutrition post‐surgery [107]. A systematic review of 33 observational studies (*n* = 14 880) found higher odds of perinatal mortality, congenital anomalies, preterm birth and neonatal intensive care unit admission [108]. Guthrie et al. [109] reported high rates of micronutrient deficiency during pregnancy despite supplementation, though study limitations prevented firm conclusions on which care aspects improve outcomes [109]. A clinical guideline provides recommendations on nutritional monitoring and supplementation during pregnancy post‐surgery, but highlights many are based on low‐quality evidence requiring further research [110].

One of the most frequently mentioned contributing factors was non‐adherence to vitamin supplements. A WHO review of evidence on adherence to long‐term therapies highlights the magnitude of the challenge of adherence, reporting adherence to long‐term therapy in high‐income countries of only 50%, with major impacts on healthcare outcomes and costs [111]. The report advocates support, not blame, a patient‐centred and multi‐disciplinary approach, and recognition of the multifactorial nature of suboptimal adherence. A narrative review by Smelt et al. of factors influencing adherence to multivitamin supplementation after bariatric surgery identified: procedure‐related complications and side‐effects, such prolonged vomiting and diarrhoea due to dumping syndrome or bacterial overgrowth; therapy‐related factors such as needing to take multiple medications and difficulty swallowing; and psychosocial factors such as social support and cost [112]. A survey by Smets et al. of 4975 patients at a Dutch bariatric centre found that 15.4% of those surveyed reported inconsistent multi‐vitamin supplementation at 7.4% no use [113]. Among inconsistent users, reasons included forgetting to take them (68.3%), gastro‐intestinal side effects (25.6%) and unpleasant taste or smell (22.7%). Non‐user reasons included gastro‐intestinal side effects (58.5%), high costs (13.5%) and the absence of vitamin deficiencies (20.9%). Overall, 28.5% were dissatisfied with medication instructions, attention paid to supplementation during medical consultations and the extent to which their personal preferences were considered. An earlier survey by Mahawar et al. involving 529 UK‐based patients found similar results, as only 45.5% reported complete compliance with supplementation with “difficulty in remembering” reported as the most important reason for poor adherence (45.6%) [13]. There is a need for in‐depth qualitative studies exploring barriers and facilitators to nutritional follow‐up and supplementation among bariatric surgery patients.

Frequently mentioned healthcare factors included missed or delayed diagnosis of nutritional deficiencies, and some cases highlighted a greater need for health professional education. The narrative review by Smelt et al. found no data on how knowledgeable health professionals are on post‐bariatric surgery medication prescribing or the role of post‐operative follow‐up visits, including psychological and behavioural support. Evaluation of the knowledge and confidence of General Practitioners in post‐bariatric surgery care would be particularly pertinent, as patients are generally discharged from specialist services to the community for follow‐up (in the UK this happens 2 years post‐operatively); yet the available evidence is scanty. GPs in Scotland (*n* = 230) surveyed in 2012 were mostly not comfortable managing patients who had undergone bariatric surgery (76%) and 30% reported a lack of awareness of national bariatric surgery guidelines [114]. Similar results were found in surveys conducted in the USA, Canada, and France [12, 115, 116]. Watkins et al. conducted semi‐structured interviews with healthcare professionals (*n* = 26, 10 of whom were GPs) exploring their experiences of long‐term post‐bariatric surgery follow‐up [117]. A lack of established care pathways was highlighted, with follow‐up often contingent on the patients' surgical pathway and the culture and expertise within the GP surgery. Suggested opportunities for improvement included using technology, adapting approaches used for other chronic conditions, shared care models, and peer support. A review of the care of 381 patients who underwent bariatric surgery in England, Wales, and Northern Ireland found that 17 received insufficient or inappropriate dietary advice, and in 189 cases it was not possible to assess whether the dietary advice given was appropriate [118]. There are national and international guidelines on nutritional support post‐bariatric surgery. Both the British Obesity and Metabolic Surgery Society (BOMSS) full nutritional guidance and guidance for GPs recommend routine monitoring of full blood count, corrected calcium, liver function, urea and electrolytes, vitamin B12 and folate, ferritin, and vitamin D [21, 119]. They recommend testing zinc, copper, and selenium if symptomatic of deficiency, or routinely if they had an SG, RYGB, or OAGB with BP limb of 150 cm or less. Vitamins E, A, and K1 are recommended if a patient has symptoms. Routine lifelong vitamin and mineral supplementation is recommended. Guidelines by Mechanik et al. in the USA provide detailed recommendations on screening for nutritional deficiencies and prophylactic supplementation [120]. EASO guidelines on post‐bariatric surgery medical management recommend involvement of experienced nutritionists and dieticians and detail recommended supplementation and routine surveillance blood tests [8].

Those working in primary care may benefit from education about presentations, signs, and symptoms of nutritional deficiencies after bariatric surgery. BOMSS have created a “GP Hub” providing information and links to detailed guidance for GPs on post‐bariatric surgery annual reviews, including a pre‐consultation patient questionnaire and GP consultation guide [121]. There is also a need for adequate funding. In the UK, there is no specific funding to support the delivery of long‐term post‐bariatric surgery follow‐up [117].

This study has some key limitations. We chose a rapid review approach, which, as described by Khangura et al. [18] can provide a streamlined approach to synthesising the evidence in a way that is timely, user‐friendly, and accessible to knowledge users. We used key aspects of systematic review methodology, including systematic searches of multiple databases, systematic screening, data extraction, and quality appraisal processes. However, in contrast to gold standard systematic review methodology [122], studies not available in English were excluded, and we were only able to double screen, double data extract, and double quality appraise 10% of included case reports, increasing the possibility of studies meeting our inclusion criteria being missed as they were not double screened, or inaccuracies in data extraction, thereby reducing reliability and consistency. Gold standard methodology, including independent double screening and data extraction, would have led to a more robust review with reduced risk of bias, but would have impaired our capacity to deliver outputs in a timely, accessible, and resource‐efficient manner. As this is a review of case reports aiming to provide a greater understanding of sequelae for real patients of nutritional deficiencies and highlight issues around follow‐up, late diagnosis, and contributory factors, this is less likely to have increased risk of bias. In view of this risk, our interpretations are more cautious, recognising the limitations of both the methodology and included study design. Case reports have limited generalisability and are at risk of publication bias [123]. However, they are useful for generating hypotheses and providing an in‐depth understanding of a patient narrative. Synthesising them in a systematic review highlights challenges with follow‐up monitoring and management of late micronutrient deficiencies after bariatric surgery and can provide evidence around some of the questions from policy and practice regarding individual patient trajectories and learning from these. Qualitative data on contributing factors and reasons for non‐adherence were not sufficiently rich for qualitative synthesis, and instead, we conducted a robust narrative synthesis according to Popay et al. [27].

In conclusion, this systematic review of case reports on late micronutrient deficiencies after bariatric surgery highlights the wide range of deficiencies that can occur; the range of ways in which these can present; and that once one deficiency is identified, more are often found. It also demonstrates that missed opportunities for earlier intervention are often identified, including appropriate nutritional counselling and support to adhere to nutritional supplements. Health professionals must be aware of the risk of harm from late nutritional deficiencies and consider these in patients whose surgery may have been many years ago and so may not, on presentation, be the most obvious cause of their symptoms.

Given that some of the deficiencies identified in the included case studies preceded or caused permanent disability or death, preventing late nutritional deficiency post‐bariatric surgery, and early identification and management of deficiencies that do occur, is key.

## Conflicts of Interest

H.M.P. is a British Obesity and Metabolic Surgery Society (BOMSS) council member and has organised educational events supported by Ethicon for BOMSS members (honoraria received). H.M.P. developed an algorithm and accompanying resources for the management of obesity in primary care which were supported by arm's length sponsorship from Novo Nordisk (honoraria received). H.M.P. is a member of the current NICE obesity management clinical guidelines and quality standards committees.

## Data Availability

Data sharing is not applicable to this article as no new data were created or analyzed in this study.

## Appendix 1 MEDLINE Search Strategy

**Table:**

1	exp. Bariatric Surgery/29009	
2	(bariatric adj3 surg*).mp. [mp = title, abstract, original title, name of substance word, subject heading word, floating sub‐heading word, keyword heading word, organism supplementary concept word, protocol supplementary concept word, rare disease supplementary concept word, unique identifier, synonyms]	19 431
3	“gastric band*”.mp.	3610
4	“sleeve gastrectom*”.mp. [mp = title, abstract, original title, name of substance word, subject heading word, floating sub‐heading word, keyword heading word, organism supplementary concept word, protocol supplementary concept word, rare disease supplementary concept word, unique identifier, synonyms]	5697
5	“duodenal switch”.mp. [mp = title, abstract, original title, name of substance word, subject heading word, floating sub‐heading word, keyword heading word, organism supplementary concept word, protocol supplementary concept word, rare disease supplementary concept word, unique identifier, synonyms]	763
6	exp. Gastric Balloon/676	
7	“gastric balloon”.mp.	795
8	exp. Biliopancreatic Diversion/1052	
9	“biliopancreatic diversion”.mp.	1441
10	“gastric bypass”.mp.	13 678
11	“nutritional deficien*”.mp. [mp = title, abstract, original title, name of substance word, subject heading word, floating sub‐heading word, keyword heading word, organism supplementary concept word, protocol supplementary concept word, rare disease supplementary concept word, unique identifier, synonyms]	4503
12	exp. Nutrition Therapy/106200	
13	“nutritional therap*”.mp. [mp = title, abstract, original title, name of substance word, subject heading word, floating sub‐heading word, keyword heading word, organism supplementary concept word, protocol supplementary concept word, rare disease supplementary concept word, unique identifier, synonyms]	2060
14	Nutritional Physiological Phenomena/28818	
15	exp. Avitaminosis/76318	
16	“vitamin deficien*”.mp.	2193
17	“nutritional support”.mp. [mp = title, abstract, original title, name of substance word, subject heading word, floating sub‐heading word, keyword heading word, organism supplementary concept word, protocol supplementary concept word, rare disease supplementary concept word, unique identifier, synonyms]	12 678
18	“nutritional correction*”.mp. [mp = title, abstract, original title, name of substance word, subject heading word, floating sub‐heading word, keyword heading word, organism supplementary concept word, protocol supplementary concept word, rare disease supplementary concept word, unique identifier, synonyms]	23
19	“nutritional treat*”.mp.	810
20	exp. Magnesium Deficiency/4335	
21	“magnesium deficien*”.mp.	4784
22	Protein Deficiency/4992	
23	“protein deficien*”.mp.	7398
24	“thiamine deficien*”.mp.	3469
25	“vitamin A deficien*”.mp.	6981
26	(iron adj1 deficien*).mp. [mp = title, abstract, original title, name of substance word, subject heading word, floating sub‐heading word, keyword heading word, organism supplementary concept word, protocol supplementary concept word, rare disease supplementary concept word, unique identifier, synonyms]	23 356
27	“vitamin B 12 deficien*”.mp.	6531
28	“vitamin B12 deficien*”.mp. [mp = title, abstract, original title, name of substance word, subject heading word, floating sub‐heading word, keyword heading word, organism supplementary concept word, protocol supplementary concept word, rare disease supplementary concept word, unique identifier, synonyms]	3002
29	“folic acid deficien*”.mp. [mp = title, abstract, original title, name of substance word, subject heading word, floating sub‐heading word, keyword heading word, organism supplementary concept word, protocol supplementary concept word, rare disease supplementary concept word, unique identifier, synonyms]	5301
30	“vitamin B deficien*”.mp. [mp = title, abstract, original title, name of substance word, subject heading word, floating sub‐heading word, keyword heading word, organism supplementary concept word, protocol supplementary concept word, rare disease supplementary concept word, unique identifier, synonyms]	1993
31	“vitamin D deficien*”.mp.	20 900
32	“vitamin K deficien*”.mp.	2569
33	“calcium deficien*”.mp.	1577
34	“zinc deficien*”.mp.	4610
35	“copper deficien*”.mp.	2121
36	“selenium deficien*”.mp.	1898
37	roux en y.mp.	12 019
38	vertical band*.mp.	819
39	1 or 2 or 3 or 4 or 5 or 6 or 7 or 8 or 9 or 10 or 37 or 38	39 491
40	Night Blindness/1479	
41	micronutrient deficien*.mp.	2090
42	Anaemia/or anaemia.mp.	74 950
43	11 or 12 or 13 or 14 or 15 or 16 or 17 or 18 or 19 or 20 or 21 or 22 or 23 or 24 or 25 or 26 or 27 or 28 or 29 or 30 or 31 or 32 or 33 or 34 or 35 or 36 or 40 or 41 or 42	327 379
44	39 and 43	2516
45	limit 44 to (english language and year =“2000 Current”)	2114
46	limit 45 to (“all adult (19 plus years)” or “young adult (19 to 24 years)” or “adult (19 to 44 years)” or “young adult and adult (19–24 and 19–44)“or “middle age (45 to 64 years)” or “middle aged (45 plus years)” or “all aged (65 and over)” or “aged (80 and over)”)	1245
